# From deep TLS validation to ensembles of atomic models built from elemental motions. II. Analysis of TLS refinement results by explicit interpretation

**DOI:** 10.1107/S2059798318005764

**Published:** 2018-06-08

**Authors:** Pavel V. Afonine, Paul D. Adams, Alexandre Urzhumtsev

**Affiliations:** a Molecular Biophysics and Integrated Bioimaging Division, Lawrence Berkeley National Laboratory, Berkeley, California, USA; b Department of Physics and International Centre for Quantum and Molecular Structures, Shanghai University, Shanghai 200444, People’s Republic of China; c Department of Bioengineering, University of California Berkeley, Berkeley, California, USA; d Centre for Integrative Biology, Institut de Génétique et de Biologie Moléculaire et Cellulaire, CNRS–INSERM–UdS, 1 Rue Laurent Fries, BP 10142, 67404 Illkirch, France; e Faculté des Sciences et Technologies, Université de Lorraine, BP 239, 54506 Vandoeuvre-les-Nancy, France

**Keywords:** TLS model, TLS refinement, atomic displacement parameters, rigid-body motion, ensemble of atomic models, atomic model validation, PDB

## Abstract

TLS modelling was developed by Schomaker and Trueblood to describe atomic displacement parameters through concerted (rigid-body) harmonic motions of an atomic group [Schomaker & Trueblood (1968 ▸), *Acta Cryst.* B**24**, 63–76]. The results of a TLS refinement are **T**, **L** and **S** matrices that provide individual anisotropic atomic displacement parameters (ADPs) for all atoms belonging to the group. These ADPs can be calculated analytically using a formula that relates the elements of the TLS matrices to atomic parameters. Alternatively, ADPs can be obtained numerically from the parameters of concerted atomic motions corresponding to the TLS matrices. Both procedures are expected to produce the same ADP values and therefore can be used to assess the results of TLS refinement. Here, the implementation of this approach in *PHENIX* is described and several illustrations, including the use of all models from the PDB that have been subjected to TLS refinement, are provided.

## Abstract 2

The values of anisotropic atomic displacement parameters (ADPs) that correspond to concerted motions can be obtained from refined TLS matrices analytically or numerically. The difference between the ADPs obtained using these two methods can be used to assess the results of TLS refinement.

## 1. Introduction   

### 1.1. Atomic positions in crystal structures   

Describing atomic positions in crystal structures by Cartesian coordinates is a mathematical abstraction. Atomic positions are averages over the diffraction data-collection time and over all of the unit cells in the crystal. The variation of positions may range from large, representing discrete conformations, to small, reflecting atomic motion around a central position.

If a motion is harmonic (in particular, this means that the motion amplitude is small), the probability of a shift of an atom *n* by a vector **r**_*n*_ = Δ*x*_*n*_ **i** + Δ*y_n_* **j** + Δ*z_n_* **k** is defined by individual isotropic (*B_n_*) or anisotropic (**U** *_n_*) atomic displacement parameters (ADPs):
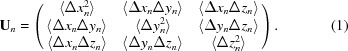
These characteristics of atomic mobility are part of the structural information that is associated with models of crystal structures. As discussed in the literature (see, for example, Dunitz & White, 1973 ▸; Murshudov *et al.*, 1999 ▸; Winn *et al.*, 2001 ▸), the atomic displacement is a superposition of various motions that arise from different sources. These include displacement of atoms as part of a group and individual vibrations. A group motion itself can have several sources such as motion of the whole molecule, motion of its domains, side-chain libration *etc*. Typically, modern refinement programs treat these motions using three separate components: motion of the whole crystal (modelled as an overall anisotropic scale factor), motion of non-overlapping groups that are considered to be rigid, and individual atomic motions.

### 1.2. Rigid-group motion   

Atomic displacements arising from rigid-body motions can be accounted for using the TLS model (Schomaker & Trueblood, 1968 ▸). Such a model is based on simple geometric considerations allowing the description of elemental harmonic motions of atomic groups in terms of three matrices **T**, **L** and **S** (for a review, see Urzhumtsev *et al.*, 2013 ▸). This provides a convenient mathematical way to present these motions in terms of an individual anisotropic ADP,
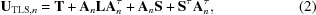
where
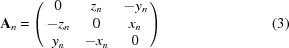
is an antisymmetric matrix expressed using the Cartesian coordinates (*x*_*n*_, *y*_*n*_, *z*_*n*_) of atom *n* with respect to the origin of the TLS group. The symbol τ denotes the matrix transpose. The TLS approach may be seen as a statistical model for the analytical averaging of atomic positions that vary according to the given elemental motion parameters. The simplest example of a common motion is an isotropic vibration of a group that is equivalent to the assignment of the same *B* value to all atoms of the group. In the TLS model, the symmetric matrix **L** corresponds to the libration of a group, the symmetric matrix **T** corresponds to its common vibrations^1^ (also including a correction for the position of the libration axes) and the matrix **S** reflects correlations between the motions as well as the position of the axes.

A set of TLS matrices is defined by 21 parameters (six for **T**, six for **L** and nine for **S**). There is a linear constraint on the diagonal elements of the **S** matrix (Schomaker & Trueblood, 1968 ▸), resulting in 20 independent parameters. If individual atomic displacements can be ignored and the assumption that atomic motions are purely rigid can be accepted (at low resolution, for example), then modelling atomic displacements using TLS can significantly reduce the overall number of fitting parameters. In the following, we refer to the parameterization of an atomic group motion using parameters of elemental rigid-body motions as direct parameterization and that using elements of the **T**, **L** and **S** matrices, such as in (2), as indirect parameterization.

Indirect parameterization is mathematically and computationally more straightforward compared with direct parameterization. This is because of the simple relationship between the refinable elements of the **T**, **L** and **S** matrices and the atomic displacement parameters **U** using (2). In contrast, direct parameterization requires a nontrivial number of mathematical steps that link the parameters of atomic motions (such as the amplitudes of vibration and libration *etc.*) to the elements of TLS matrices (see, for example, Urzhumtsev *et al.*, 2015 ▸). It is thus unsurprising that model-refinement programs such as *phenix.refine* (Afonine *et al.*, 2012 ▸) and *REFMAC* (Murshudov *et al.*, 1997 ▸) use indirect parameterization for TLS owing to its simplicity; that is, they refine the elements of TLS matrices and not the actual parameters of atomic motions. This approach is inherently problematic because unconstrained or unrestrained refinement of TLS matrices does not guarantee that the derived parameters of atomic motions are physically realistic or comply with TLS theory (see, for example, Zucker *et al.*, 2010 ▸; Merritt, 2012 ▸; Urzhumtsev *et al.*, 2015 ▸). This is very similar to unrestrained refinement of atomic coordinates at typical ‘macromolecular resolutions’ (*e.g.* 2–3 Å): factually, such refinement would almost certainly result in distorted stereochemistry.

### 1.3. Two possible interpretations of TLS models   

One may think of at least two possible ways to interpret the results of TLS refinement. One interpretation considers TLS modelling to be successful if it leads to an improvement in the *R* factors and if the atomic displacement parameters **U**_TLS,*n*_ derived from the refined TLS matrices using (2) are realistic (for example, they vary smoothly between neighbouring atoms). A more conservative approach considers TLS modelling to be successful if, in addition to meaningful ADPs and improved model-to-data fit, the TLS parameters comply with the basic assumptions of the corresponding theory set out by Schomaker & Trueblood (1968 ▸). This additional requirement is important when atomic motions modelled using TLS parameters are used to describe molecular motions (Trueblood, 1978 ▸; Trueblood & Dunitz, 1983 ▸; and references therein) or diffuse X-ray scattering data (Van Benschoten *et al.*, 2015 ▸), or are analysed for biological significance (Kuriyan & Weis, 1991 ▸; Harris *et al.*, 1992 ▸; Šali *et al.*, 1992 ▸; Wilson & Brunger, 2000 ▸; Raaijmakers *et al.*, 2001 ▸; Yousef *et al.*, 2002 ▸; Papiz *et al.*, 2003 ▸; Chaudhry *et al.*, 2004 ▸); see also discussion in Merritt (1999 ▸).

### 1.4. Analytical and numerical calculations of ADPs from TLS models   

An interpretation of TLS refinement results in terms of elemental motions (see, for example, Howlin *et al.*, 1993 ▸; Urzhumtsev *et al.*, 2015 ▸, 2016 ▸) provides an opportunity to verify whether the corresponding motion parameters agree with TLS theory. This can be performed in two steps as follows. Firstly, the parameters of elemental group motions extracted from refined TLS matrices can be used to obtain an ensemble of models that samples these motions. In turn, (1) can be used to convert the ensemble back to a single model with the uncertainties in atomic positions described using the corresponding ADP values, **U**_ensemble,*n*_. Secondly, (2) can be used to calculate the uncertainties **U**_TLS,*n*_ in atomic positions directly from the TLS matrices. It is intuitive to expect that **U**_TLS,*n*_ and **U**_ensemble,*n*_ will match within some tolerance. The tolerance is needed to account for rounding errors and the finite number of models in the ensemble. A difference between **U**_TLS,*n*_ and **U**_ensemble,*n*_ beyond this tolerance may be indicative of various problems with the corresponding TLS set.

**Figure 1:**
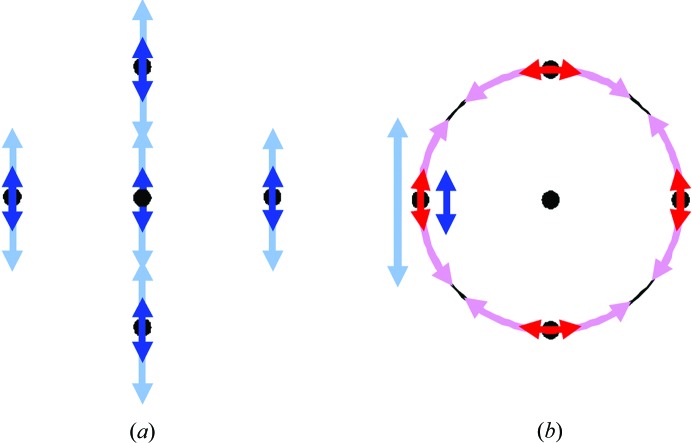
(*a*) A schematic representation of the atomic displacement for pure vibrations along the vertical axis (light and dark blue arrows) and (*b*) for libration around the axis perpendicular to the view (light and dark red arrows) shown for a five-atom dummy model (black dots). Lighter coloured arrows correspond to displacements with larger amplitudes. The displacements for vibration and libration are similar for small amplitudes and different for large amplitudes (*b*). The curvature of libration displacements with large amplitudes (*b*) makes them anharmonic.

Since currently used refinement programs utilize an indirect TLS parameterization that does not use restraints or constraints, it may be the case that extracting motion parameters from refined TLS matrices is mathematically impossible (Urzhumtsev *et al.*, 2015 ▸, 2016 ▸). The simplest example is the **T** or **L** matrices being non-positive definite. A more subtle example is when the parameters of elemental motions can be extracted from the TLS matrices but may not satisfy the basic assumptions about the TLS model (for example, libration amplitudes being too large, resulting in atomic motions that are anharmonic; see Fig. 1 ▸ and §[Sec sec2]2).

When motion parameters can be extracted from TLS matrices, comparison of **U**_ensemble,*n*_ and **U**_TLS,*n*_ requires a measure of and a threshold for the tolerance mentioned above (discussed in §[Sec sec2.1]2.1). Since **U**_ensemble,*n*_ depends on the number of models in the ensemble, we use a simple test system to estimate how many models are required to sample the group motion accurately and also to estimate a possible threshold value for the similarity of respective matrices (§[Sec sec2.2]2.2). The results are then validated using a more realistic protein model (§[Sec sec2.3]2.3). These tests highlighted reasons for differences between **U**_TLS,*n*_ and **U**_ensemble,*n*_ matrices and prompted further improvements for TLS analysis (§[Sec sec2.4]2.4). In §[Sec sec3]3 we discuss the results of the application of our procedures to all models in the PDB (Bernstein *et al.*, 1977 ▸; Berman *et al.*, 2000 ▸) that contain TLS information. Respective tools have been added to the *PHENIX* suite (Adams *et al.*, 2010 ▸).

## 2. Anisotropic displacement matrices and the corresponding model ensembles   

### 2.1. Metrics for matrix comparison   

To evaluate the similarity of the two sets of anisotropic displacement matrices, **U**_TLS,*n*_ and **U**_ensemble,*n*_, for a group composed of *N* atoms, *n* = 1, 2, …, *N*, we arbitrarily choose to use a simple *R*-factor-type metric,
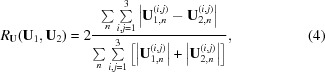
where **U**_1,*n*_ = **U**_TLS,*n*_ and **U**_2,*n*_ = **U**_ensemble,*n*_. Here, the sums are calculated over all elements of the matrices and over all atoms of the group. Other metrics can also be used (Dunitz & White, 1973 ▸; Zucker *et al.*, 2010 ▸). Specifically, Kullback–Liebler (KL) divergence (Kullback & Leibler, 1951 ▸; Murshudov *et al.*, 2011 ▸; Merritt, 2011 ▸, 2012 ▸) and the correlation coefficient (CC_**UV**_; Merritt, 1999 ▸) seem to be most prominent, with the caveat that they require matrix inversion, which is not always possible in numeric tests where only one single motion can be considered.

The calculation of (4) depends on the randomly generated ensemble models that are used to obtain **U**_ensemble,*n*_. This is a stochastic procedure that depends on random seed values and on the number of models in the ensemble. Below, we analyze how these parameters affect the estimate of **U**_ensemble_. Also, we check whether using KL_**UV**_ or CC_**UV**_ leads to conclusions that differ from those obtained using *R*_**U**_.

### 2.2. Illustrations using a one-atom model   

For simplicity, in this section we drop the subscript *n* from **U**_ensemble_ and **U**_TLS_ because only a single atom is considered.

#### 2.2.1. Effect of vibration   

**Figure 2:**
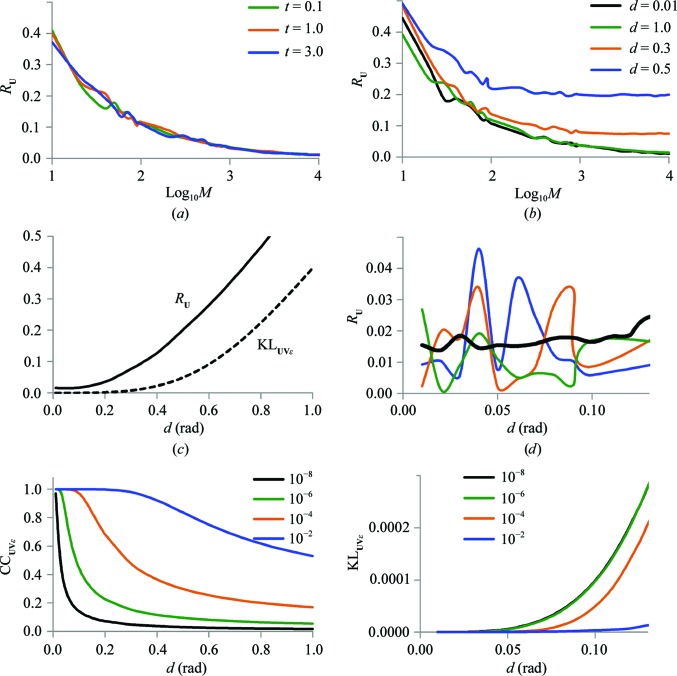
Agreement between the **U**_ensemble_ and **U**_TLS_ matrices calculated for a single-atom model. *R*_**U**_ (averaged over 100 random runs) is shown as a function of the logarithm of the number *M* of models for different (*a*) vibration and (*b*) libration r.m.s.d. values. (*c*) *R*_**U**_ (solid line) and KL_**UV**∊_ (dashed line) with ∊ = 10^−6^ as a function of the vibration r.m.s.d. value *d* for ensembles composed of 5000 generated models. (*d*) *R*_**U**_ as a function of the vibration r.m.s.d. value *d* zoomed on the *d* = 0.0–0.1 rad range and shown for the average (black curve) as well as for three individual runs (in maroon, blue and green) selected from the 100 runs used for averaging. (*e*) CC_**UV**∊_ calculated for several ∊ values (10^−2^, 10^−4^, 10^−6^ and 10^−8^). (*f*) KL_UV∊_ calculated for the same ∊ values and for small *d* values; the curves for ∊ values of 10^−6^ and 10^−8^ are indistinguishable. See the text for details.

In this test, we consider a model composed of a single atom vibrating along the **Ox** axis. For each trial root-mean-square deviation (r.m.s.d., which we call the vibration amplitude *t*), we generated *M* random copies of this atom and then took all of these copies to calculate **U**_ensemble_ using (1). We then used (4) to compare **U**_ensemble_ with the corresponding **U**_TLS_ = **T** calculated analytically using (2) with **L** = **S** = 0. For each trial *t* we repeated these calculations 100 times, each time with a different random seed. Obviously, for different trials **U**_TLS_ remains the same while **U**_ensemble_ varies. Fig. 2(*a* ▸) shows the average (over 100 trials) *R*_**U**_ for different trial values of *t* and *M*. The results are essentially independent of *t*. This is expected since 〈Δ*x* ^2^〉 in (1) is proportional to *T*_*xx*_ = *t*_*x*_^2^ in (2) for a sufficiently large number of models. We observe that *R*_**U**_ becomes close to 0.01 once the size of the ensemble reaches about 10 000 models.

#### 2.2.2. Effect of libration   

Here, we used the same single-atom model as above and the same calculation workflow, except that we varied the libration r.m.s.d. value *d*. Similarly to the previous example, *R*_**U**_ as a function of ensemble size reaches a plateau for about 5000–10 000 models in the ensemble (Fig. 2 ▸ *b*); however, the plateau level depends on the value of *d*. Then we sampled a broad range of *d* values keeping the ensemble size fixed at 5000 models (Figs. 2 ▸ *c* and 2 ▸ *d*). We observe that *R*_**U**_ remains approximately constant (∼0.02) up to a *d*_0_ of ∼0.15 rad and then starts increasing monotonically. The *d*_0_ value obtained in this numerical experiment corresponds to the limit of a linear approximation to the small rotations discussed in Urzhumtsev *et al.* (2013 ▸) and other works cited therein, for example Cruickshank (1956 ▸). Owing to rounding errors and the differences between a rotation motion and a linear motion, the *R*_**U**_ values never reached zero even for very small *d* and large ensemble sizes (Fig. 2 ▸ *c*). Also, while the average values over several trials are stable, they may vary between individual trials (Fig. 2 ▸ *d*). These results allowed us to draw two conclusions. Firstly, generating about 5000–10 000 models is sufficient to estimate **U**_ensemble_ reliably (in §[Sec sec2.3]2.3 we show that this is still the case for realistic macromolecular models). Secondly, we may consider that **U**_ensemble_ agrees with **U**_TLS_ for a particular TLS set if *R*_**U**_ is approximately 0.05 or less.

#### 2.2.3. Checking other metrics   

To illustrate that the results obtained in previous tests are independent of matrix-comparison metrics, we repeated the test described in §[Sec sec2.2.2]2.2.2 using other metrics such as KL_**UV**_ and CC_**UV**_. Since these metrics require a matrix inversion, we had to use a minor modification consisting of adding a small value to all of the diagonal elements of the respective matrices **U**_ensemble_ and the corresponding **U**_TLS_, which is a convolution with an isotropic vibration. For example, the modified KL_**UV**_ metric is KL_**UV**∊_ = ∊tr(**U**_∊_ **V**_∊_^−1^ + **V**_∊_ **U**_∊_^−1^ − 2**I**) with **U**_∊_ = **U** + ∊**I** and **V**_∊_ = **V** + ∊**I**. The scale factor *∊* before ‘tr’ is used to put the results on a similar scale (to facilitate comparisons). By trial and error, we found that a value of ∊ in the range 10^−8^–10^−6^ allows the calculation of KL_**UV**∊_ and CC_**UV**∊_ but does not significantly affect the results. Overall, KL_**UV**∊_, CC_**UV**∊_ and *R*_**U**_ do not contradict each other (Fig. 2 ▸ *c*), with CC_UV∊_ showing a much stronger dependence on ∊ (Fig. 2 ▸ *e*) and both KL_**UV**∊_ and CC_**UV**∊_ showing a less prominent drop (Figs. 2 ▸ *e* and 2 ▸ *f*) at a *d*_0_ of ∼0.10–0.15 rad (see Cruickshank, 1956 ▸) compared with *R*_U_. In the following we use *R*_**U**_ because the original matrices can be used without modification and it has a predictable range of values, unlike KL_**UV**∊_.

### 2.3. Illustrations using a protein model   

As a more realistic example, we selected the model of IgG-binding domain III (PDB entry 2igd; S. Butterworth, V. L. Lamzin, D. B. Wigley, J. P. Derrick & K. S. Wilson, unpublished work) refined at 1.1 Å resolution using individual anisotropic ADP values. We chose the core of this model as a single TLS group containing residues 6–61 (leaving out the flexible N-terminus).

We considered two models derived from these data. One model contained C^α^ atoms only (56 in total) and the other model included all main-chain atoms (C^α^, O, C and N). Each of the two models was treated as a single TLS group. For each model we fitted TLS matrices to individual anisotropic **U**_*n*_ values (ANISOU records from the PDB file) using the *phenix.tls* tool; we refer to these matrices as TLS_CA_ and TLS_MC_, respectively. Then, using each of the two TLS sets (TLS_CA_ and TLS_MC_) we calculated **U**_TLS,*n*_ using (2) and generated **U**_ensemble,*n*_ as described in Urzhumtsev *et al.* (2015 ▸). Similarly to as described in §[Sec sec2.2]2.2, we sampled a range of different numbers of models per ensemble.

**Figure 3:**
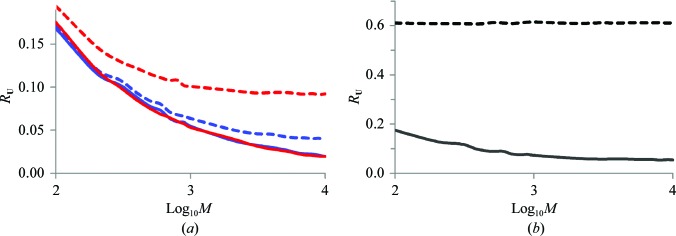
Agreement of *R*_**U**_ between **U**_ensemble_ and **U**_TLS_ matrices as a function of the number of generated models calculated for protein data. (*a*) Results for 2igd models composed of all main-chain atoms (red) and C^α^ atoms only (blue) using different approaches to extract the elemental motions: dashed lines for (10) and full lines for (11). (*b*) Results for the 4muy model using (10) shown as a dashed line and (11) shown as a full line.

The blue dashed curve in Fig. 3 ▸(*a*) shows that for the C^α^-only model (TLS_CA_) *R*_**U**_ becomes smaller than 0.05 when the ensemble contains about 5000 models; using a larger ensemble does not change *R*_**U**_ significantly. This agrees with the conclusions derived in §[Sec sec2.2]2.2. For the main-chain model *R*_**U**_ reaches a plateau for ensembles containing the same number of models, but the value of *R*_**U**_ does not decrease below 0.09 (red dashed curve in Fig. 3 ▸ *a*). To investigate the source of such a significant difference in *R*_**U**_ we performed the following tests.

**Figure 4:**
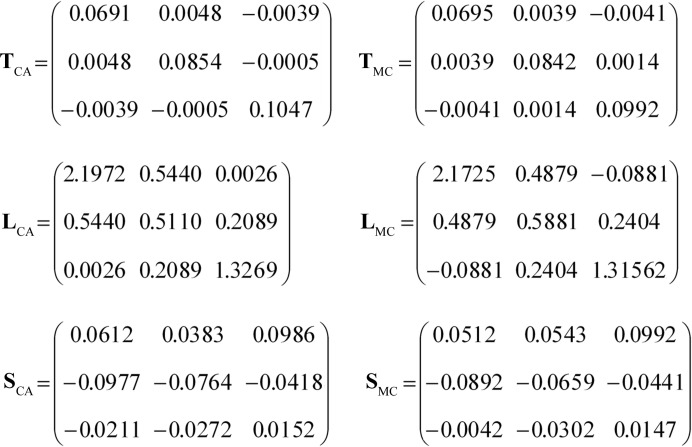
The TLS matrices calculated for the 2igd model for all main-chain atoms (right) and for C^α^ atoms only (left). The matrices are given according to the PDB conventions: **T** is in Å^2^, **L** is in deg^2^ and **S** is in Å deg.

Firstly, we note that the only difference between the two models is their composition and TLS matrices (Fig. 4 ▸). To determine which of the two, the composition or TLS matrices, contributes to the large *R*_**U**_ value, we repeated the calculations above using the C^α^-only model with TLS_MC_ matrices and the main-chain model with TLS_CA_ matrices. In the first case *R*_**U**_ was 0.09 and in the second case it was 0.05. This shows that the difference in *R*_**U**_ is owing to the TLS matrices and is not owing to the model composition. To find out which features of TLS_MC_ are responsible for the increased *R*_**U**_, we performed a further analysis.

The elemental motions encoded by TLS matrices are three screw librations (around three mutually orthogonal axes **l**_*x*_, **l**_*y*_, **l**_*z*_) coupled with three vibrations, also about three mutually orthogonal axes **v**_*x*_, **v**_*y*_, **v**_*z*_. In the following, *d_x_*, *d_y_*, *d_z_* stand for libration amplitudes, *t_x_*, *t_y_*, *t_z_* stand for vibration amplitudes, *s_x_*, *s_y_*, *s_z_* stand for the corresponding screw parameters and **w**_*x*_, **w**_*y*_, **w**_*z*_ stand for the points that belong to the respective libration axes (for formal definitions, see Urzhumtsev *et al.*, 2013 ▸). As discussed in §[Sec sec2.2]2.2, the condition **U**_ensemble,*n*_ ≃ **U**_TLS,*n*_ (for a sufficiently large number of models in the ensemble) may break down owing to inadequate librations and not owing to vibrations. To separate the contribution of vibrations and librations, we first derived the set of parameters of elemental motions
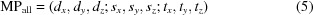
from the corresponding TLS matrices as described in Urzhumtsev *et al.* (2015 ▸). Here, and in the following, to simplify the text we drop the parameters **l**_*x*_, **l**_*y*_, **l**_*z*_; **w**_*x*_, **w**_*y*_, **w**_*z*_; **v**_*x*_, **v**_*y*_, **v**_*z*_ from the list in (5) since they are invariant within these tests. Then, using the parameters in (5) (Table 2 ▸) and the C^α^-only model, we calculated **U**_ensemble,*n*_ and **U**_TLS,*n*_ for the following different scenarios.

Firstly, we considered a scenario where all three librations are used together, including their screw components, while vibrations are excluded:
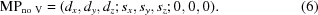
Excluding vibrations led to an increase in *R*_**U**_ for both TLS_MC_ and TLS_CA_; the values in the *R*_**U**_(no V) column in Table 1 ▸ are ∼1.5 times larger^2^ than those for *R*_**U**_(all).

Secondly, we calculated *R*_**U**_ separately for each individual libration, including its corresponding screw component (Table 1 ▸, columns 5–7),
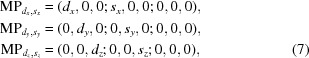
and without it,
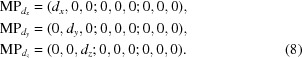
(Table 1 ▸, columns 8–10). For the screw librations, *R*_**U**_ is large for the rotation around **l**_*x*_ (Table 1 ▸), which is likely to be owing to a large magnitude of the screw component *s_x_*. This component is two and a half times greater for TLS_MC_ compared with TLS_CA_ (−5.70 *versus* −2.07; Table 2 ▸), resulting in an about twofold larger *R*_**U**_ value. Removing all screw components results in an *R*_**U**_ of the order of 0.01 for all librations [*R*_**U**_(*d_x_*), *R*_**U**_(*d_y_*) and *R*_**U**_(*d_z_*) columns in Table 1 ▸].

These tests let us draw two conclusions. Firstly, the ensemble size required for reliable calculation of **U**_ensemble,*n*_ does not depend on the model size and, similarly to the one-atom case (§[Sec sec2.2]2.2), 5000–10 000 models are sufficient. Secondly, large values of the screw components are responsible for the disagreement between **U**_ensemble,*n*_ and **U**_TLS,*n*_ and the large reesulting *R*_**U**_. This conclusion prompted us to revisit the TLS decomposition algorithm described in Urzhumtsev *et al.* (2015 ▸).

### 2.4. Improvement of the TLS decomposition   

In the decomposition of the TLS matrices into elemental motions, some parameters, including libration amplitudes and axes, are defined unambiguously. However, the screw parameters are derived using the **S** matrix, which is not unique but is defined with an arbitrary constant σ that can be added to or subtracted from its diagonal elements (Schomaker & Trueblood, 1968 ▸). This freedom in the definition of **S** does not change the ADP and provides the possibility for alternative (and possibly better) decompositions of the TLS matrices. In Urzhumtsev *et al.* (2013 ▸) we discussed a possible argument for the traditional choice of σ from the condition
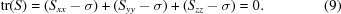
Here *S_xx_*, *S_yy_*, *S_zz_* are the diagonal elements of the matrix **S** expressed in the basis [**L**] of the principal libration directions; these directions are eigenvectors of the matrix **L**. In Urzhumtsev *et al.* (2015 ▸) we showed that (9) may result in TLS matrices that do not correspond to elemental motions, and to address this issue we suggested a better choice for the *t* value, 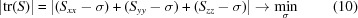
under some additional constraints on σ that are discussed in that paper.

As shown in the previous paragraph, excessively large screw parameters lead to significant discrepancies between **U**_ensemble,*n*_ and **U**_TLS,*n*_. This suggests that a better alternative to (9) and (10) might be to choose σ such that it minimizes the norm of the screw vector |**s**|. The new condition is then
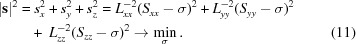
Here, according to equations (5) and (8) in Urzhumtsev *et al.* (2015 ▸), *S_xx_* − σ = *s_x_*〈*d_x_* ^2^〉 and *L_xx_* = 〈*d_x_* ^2^〉 (and similar expressions for the four other terms) are the diagonal elements of the matrices **S** and **L** given in the basis [**L**].

In order to test the new approach for adjusting the **S** matrix, we used the same models and sets of TLS matrices as described in §[Sec sec2.3]2.3. For each set of matrices, TLS_MC_ or TLS_CA_, we extracted elemental motions using (11), generated **U**_ensemble,*n*_ using corresponding models and then computed *R*_**U**_ values using the previously obtained **U**_TLS,*n*_. Table 1 ▸ shows that the updated *R*_**U**_ values calculated using (11) to adjust **S** are acceptably low not only for the total motion but for each of the individual components, both for TLS_CA_ and for TLS_MC_. Fig. 3 ▸(*a*) shows *R*_**U**_ plots as a function of the number of generated models. The curves are nearly identical for both models, showing even lower values for *R*_**U**_ than the original curve for the C^α^-only model.

**Figure 5:**
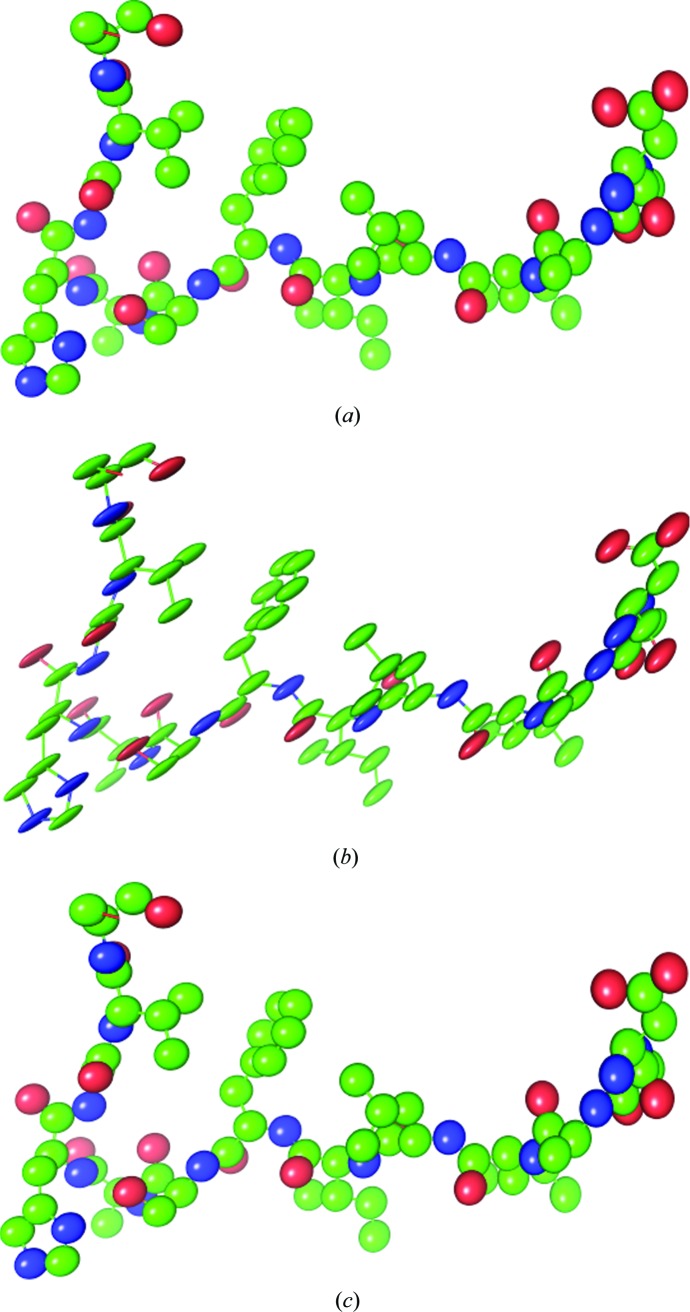
The **U** ellipsoids shown with *PyMOL* (DeLano, 2002 ▸) for the atoms of the sixth TLS group of the 4muy model. (*a*) **U**_TLS_ matrices. (*b*) **U**_ensemble_ matrices calculated with the elemental motions obtained using (10). (*c*) **U**_ensemble_ matrices calculated with the elemental motions obtained using (11).

**Figure 6:**
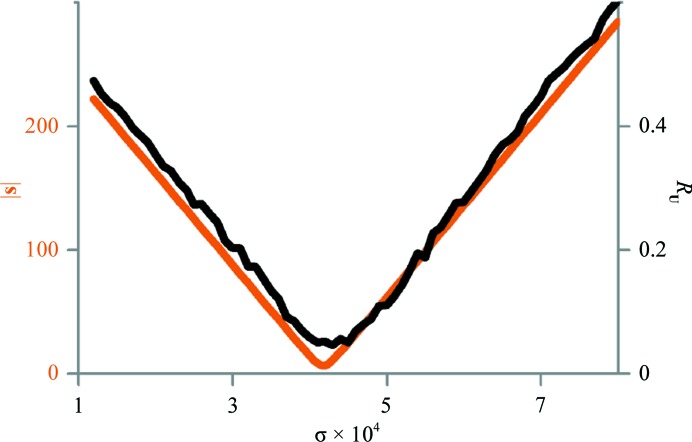
Variation of the vector norm |**s**| (11) (maroon) and of the *R*_**U**_ value (black) as a function of the parameter σ that is subtracted simultaneously from all diagonal elements of the **S** matrix during the decomposition of TLS matrices into parameters of elemental motions (4muy data; see §[Sec sec2.4]2.4). Small oscillations in *R*_**U**_ illustrate its stochastic nature.

A more striking result is obtained when applying the new correction method to a real-life example: PDB entry 4muy (Span *et al.*, 2014 ▸). In all tests TLS groups were used as defined in the PDB file. The 4muy model is composed of 40 TLS groups, and we focus on group No. 6 (residues 65–77 in chain *A*). The decomposition of the reported TLS matrices into motion parameters using the approach described previously (Urzhumtsev *et al.*, 2015 ▸) suggests removing σ = 10^−5^ from the diagonal elements of the **S** matrix (expressed in Å rad). The *R*_**U**_ corresponding to these matrices is very high at 0.61, indicating a large disagreement between **U**_ensemble,*n*_ and **U**_TLS,*n*_ (Figs. 5 ▸ *a* and 5 ▸ *b* and the dashed curve in Fig. 3 ▸ *b*). We suspected that this disagreement was owing to a very large value of the screw parameter *s_x_* of 303.6 for the screw rotation around the axis **l**_*x*_ (Table 1 ▸). Applying (11) to adjust the **S** matrix resulted in σ increasing to 42 × 10^−5^, which in turn reduced *s_x_* to 0.1 and also reduced the respective *R*_**U**_ from 0.89 to 0.02. Fig. 5 ▸(*c*) shows the thermal ellipsoids obtained using the screw libration parameters extracted with (11): clearly, **U**_ensemble,*n*_ and **U**_TLS,*n*_ are much more similar (compare with Fig. 5 ▸ *a*). Fig. 6 ▸ shows the variation of *R*_**U**_ and of |**s**| as a function of the σ value; indeed, the minimum of *R*_**U**_ is observed for σ obtained using (11). The *R*_**U**_ for the overall motion decreased to an acceptable value of 0.05, and for the libration alone it decreased from 0.85 to 0.11. The latter value is still high, possibly because by reducing *s_x_* the procedure increased the magnitudes of *s_y_* and *s_z_* (from 2.90 to −3.38 and from −3.11 to −5.14, respectively; Tables 1 ▸ and 2 ▸). This test shows both the advantage of the new approach (11) compared with (9) and (10) and also its limitations. In this test using other norms, in particular max{|*s_x_*|, |*s_y_*|, |*s_z_*|}, in (11) did not improve the result. In general, there is no guarantee that (11) always results in the best screw parameters and further improvements may be needed, for example by using a local search around the σ value obtained with (11).

Fig. 3 ▸(*b*) shows *R*_**U**_ as a function of the ensemble size for the 4muy model generated using parameters obtained with (10) and (11). It shows the significant difference between the results of the two approaches for correcting the **S** matrix and also confirms the previous observation that 5000–10 000 models are sufficient.

## 3. PDB analysis and improvement of the TLS decomposition   

### 3.1. Model selection and analysis setup   

The PDB (as of 14 November 2016) contains 123 954 entries, of which 32 162 contain TLS records. Since each PDB entry may contain more than one TLS record, a total of 260 353 TLS groups are available in the PDB. For each of these groups we tried to determine the corresponding elemental motions. This was performed using *phenix.tls_as_xyz* as described in Urzhumtsev *et al.* (2015 ▸).

88 697 groups could be interpreted in terms of elemental motions. In 263 of these cases all three matrices were composed of zeros. Some further 314 groups were excluded because the deposited TLS information was corrupted in a number of different ways (missing TLS group origin, non-interpretable atomic or TLS records *etc.*).

The remaining 88 120 TLS sets were subjected to three independent rounds of decomposition into elemental motions, each applying corrections to the **S** matrix using (9), (10) and (11), respectively. When using (10) and (11) the constraints on σ described in Urzhumtsev *et al.* (2015 ▸) were applied. For each set of extracted parameter values we analyzed the following motions.

(i) A combination of three screw rotations and the vibration component, *i.e.* the overall motion (5).
(ii) A combination of the screw rotations with no vibration components (6).
(iii) Each of the three screw rotations individually (7).
(iv) Each of the three pure rotations (8).

For each of these motions we calculated **U**_TLS,*n*_. We then generated an ensemble of 5000 models using *phenix.tls_as_xyz* and we used this ensemble to calculate **U**_ensemble,*n*_. Finally, we compared **U**_ensemble,*n*_ with **U**_TLS,*n*_ using *R*_**U**_. Details of this analysis are given in Table 3 ▸ and are commented on below.

### 3.2. Analysis of the elemental motions using (9)   

Table 3 ▸ shows the overall statistics and the number of TLS groups with *R*_**U**_ ≤ 0.05. For the overall motion combining all elemental components together (5), about half of the TLS groups for which we could extract the motion parameters pass the test condition *R*_**U**_ ≤ 0.05 (this is approximately 17% of the total number of deposited TLS groups). The same condition applied when considering libration components only (6) reduced the number of acceptable groups roughly by half. In the case of considering librations individually (equations 7 and 8) the criterion *R*_**U**_ ≤ 0.05 selects only 2.3% of the TLS groups (6107 groups).

There are 45 sets where using pure rotations gives *R*_**U**_ > 0.05, all of which correspond to large libration amplitudes. Thus, the main source of the discrepancies between **U**_ensemble,*n*_ and **U**_TLS,*n*_ are the screw components.

**Figure 7:**
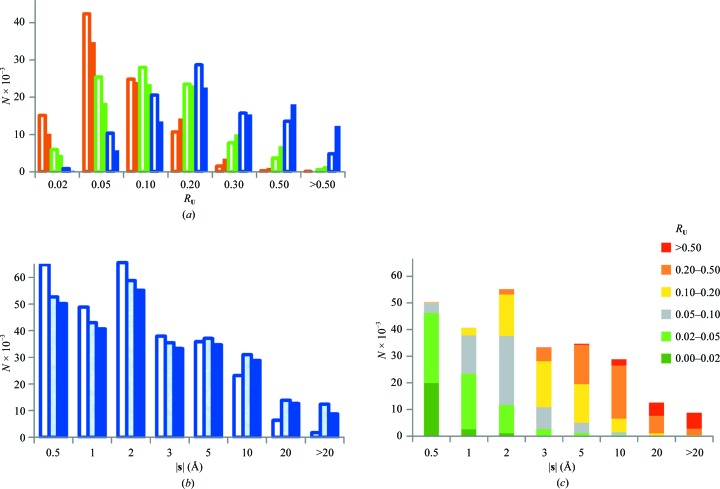
Distribution of TLS groups in the PDB. (*a*) Number of TLS groups with *R*_**U**_ values in the given intervals; distributions are shown for the total motions (maroon), for the total motions excluding vibration components (green) and for the individual screw rotations (blue). The histograms are shown when using (9) (full rectangles) and (11) (open rectangles). (*b*) Number of screw rotations as a function of the screw parameter |**s**|; the histograms are shown when using (9) (blue rectangles), (10) (light blue rectangles) and (11) (open rectangles). *R*_**U**_ values are calculated for all independent screw librations (7). (*c*) Number of TLS groups with different *R*_**U**_ values for the given interval of |**s**|. The screw parameters were extracted by the procedure using (9); *R*_**U**_ values are calculated as in (*b*). See §[Sec sec3]3 for details.

We checked (Fig. 7 ▸ *a*) the distribution of the TLS groups as a function of *R*_**U**_ calculated for the overall motion (5), for the motion excluding vibrations (6) and separately for the screw components (7). The first distribution (maroon full rectangles) has a peak in the interval 0.02–0.05 which corresponds to the TLS matrices that comply with the underlying study. Nevertheless, for a significant number of sets this value is above 0.05. Major problems come from screw components, for which many TLS groups have an *R*_**U**_ above 0.10 or even above 0.20 (blue full rectangles).

The largest value of |**s**| observed across all TLS groups is greater than 1000 Å. Such a large value means that for a rotation of 0.01 rad, *i.e.* approximately 0.6°, the rotated atoms would move by 10 Å in the direction of the rotation axis, which is clearly physically unrealistic. Fig. 7 ▸(*b*) shows that there are many groups with large values of |**s**|. The larger the screw parameter |**s**|, the larger the *R*_**U**_ values (Fig. 7 ▸ *c*). However, since a particular screw motion also depends on the libration amplitude and on the positions of the axes, this does not allow anharmonic rotations to be discriminated unambiguously using this value alone (Fig. 7 ▸ *c*).

### 3.3. Analysis of the elemental motions using (10) and (11)   

Using the approach in (10) allows motion parameters to be extracted for 6700 more TLS sets compared with (9). Table 3 ▸ and Fig. 7 ▸(*b*) show that the distributions of *R*_**U**_ values and the screw parameters |**s**| are similar to those using (9).

Repeating the same calculations using (11) shows a significantly greater difference compared with using (10) (Table 3 ▸). Considering all motions together, the number of groups for which *R*_**U**_ ≤ 0.05 increased results in more than 12 000 groups compared with using (9). Considering only screw librations, the number of groups satisfying the condition *R*_**U**_ ≤ 0.05 is doubled compared with using (9) (‘Individual screw’ column in Table 3 ▸). Fig. 7 ▸(*b*) shows that the number of rotations with a large value of the screw parameter |**s**| is significantly reduced. The largest value of |**s**| fell to below 700 Å (which is still overly large).

Fig. 7 ▸(*a*) shows that using the approach in (11) instead of that in (9) significantly shifts all three distributions to the left (compare the open rectangles with the full rectangles in Fig. 7 ▸ *a*), *i.e.* it improves the similarity between **U**_ensemble,*n*_ and **U**_TLS,*n*_. In particular, *R*_**U**_ ≤ 0.10 for the majority of TLS sets when analyzing only the matrices for the total motion (5). However, considering vibrations alone, *R*_**U**_ > 0.10 for more than a third of the models even when using the improved decomposition into elemental motions (11).

## 4. Discussion   

Validation of atomic models is now routine in macromolecular crystallography and is an integral part of structure submission to the Protein Data Bank (Read *et al.*, 2011 ▸; Gore *et al.*, 2017 ▸). It requires nomenclature compliance and fit to experimental data. Atomic coordinates are subjected to validation that includes analysis of stereochemistry and molecular packing. Atomic displacement parameters (ADPs) are also subjected to validation. For isotropic ADPs the existing validation criteria are rather simple: their values must be positive, not excessively large and not vary too much between neighbouring atoms. For anisotropic ADP values the criteria are somewhat more complex (Hirshfeld, 1976 ▸; Schneider, 1996 ▸). Similarly to atomic coordinates and displacement parameters, TLS matrices are model parameters and therefore should be subjected to some form of validation. Depending on the accepted paradigm (§[Sec sec1.3]1.3) the scope of TLS validation may refer to two questions: (i) how well does the the TLS approximation explain the experimental data and how well does it describe the atomic displacement parameters (see, for example, Merritt, 2011 ▸, 2012 ▸) and (ii) are the particular descriptors of the TLS model also consistent with the TLS formalism in addition to (i). Addressing the first question does not require analysis of the TLS matrices themselves but only of the derived ADP values. This includes making sure that the ADPs are positive definite and vary smoothly between adjacent atoms and TLS groups (Winn *et al.*, 2001 ▸; Zucker *et al.*, 2010 ▸; Merritt, 2011 ▸, 2012 ▸). The current work addresses the second question, which focuses exclusively on the analysis of TLS matrices and the parameters of group motion that they encode. Since modern atomic model refinement packages use an indirect TLS parameterization (§[Sec sec1.3]1.3), *i.e.* they refine the elements of the TLS matrices and not the parameters of group motions, it is unsurprising to find that some TLS matrices do not comply with the assumption of harmonic motion that the TLS modelling theory is built upon. The number of such cases may vary based upon the different measures or thresholds that are used. For example, using the criteria discussed above we find that only 2.3% of the TLS groups reported in the PDB can be interpreted in terms of elemental harmonic motions. We envisage two reasons for this. Firstly, the validation of TLS refinement results, focusing on TLS matrices and corresponding group motions, has never been enforced. Secondly, the implementation of TLS refinement in modern refinement packages does not allow control of the parameters of group motion by means of restraints or constraints (see a discussion in Painter & Merritt, 2006 ▸) because these parameters are refined indirectly. Unsurprisingly, such unrestrained refinement provides no guarantee of TLS matrices that are interpretable in terms of harmonic elemental motions.

In this work, we have developed methods and a software implementation in the *PHENIX* suite to analyze the results of TLS refinements. These methods are based on comparison of individual atomic displacement parameters calculated ana­lytically from the TLS matrices with ADPs derived numerically using parameters of elemental motions extracted from the TLS matrices. We theorise that large differences between these matrices indicate problematic TLS parameters. In particular, this may indicate a suboptimal choice of TLS groups or refinement protocol. We show that a post-refinement correction of the deposited TLS matrices makes it possible to curate some but not all of the problematic TLS groups.

The analyses presented in this work rely on the choice of particular criteria (metrics and thresholds). These criteria may be optimized further, which is a nontrivial project and may help to diagnose the problem while still not addressing it. A possibly better investment of effort would be to improve TLS refinement protocols so that they operate in terms of elemental parameters of motions, which has been proposed previously (Tickle & Moss, 1999 ▸). This would make it possible to control the refinable parameters directly during refinement and therefore keep them physically realistic without the need for post-refinement corrections. This is a major undertaking both mathematically and algorithmically, which may be considered as a future improvement to the *PHENIX* refinement software.

The methods and tools discussed in this manuscript have been implemented and are available in *PHENIX* 1.12 and later. Data and scripts that can be used to reproduce the figures and tables are available at http://phenix-online.org/phenix_data/afonine/tls2/.

## Figures and Tables

**Table 1:** Analysis of the discrepancy between **U**_ensemble,*n*_ and **U**_TLS,*n*_ using *R*_**U**_ For PDB entry 2igd, the two TLS sets, referred to as TLS_CA_ and TLS_MC_, are derived from anisotropic ADPs of C^α^ atoms only or of main-chain atoms, respectively. For each of the sets the parameters of the elemental motions were determined using either (10) or (11) with the constraints described in Urzhumtsev *et al.* (2015 ▸). For both TLS sets the same model composed of C^α^ atoms only was used to generate **U**_ensemble,*n*_ and compare it with the respective **U**_TLS,*n*_. For the 4muy model all atoms are used both to determine the TLS matrices and to generate **U**_ensemble,*n*_; the elemental motions were determined using either (10) or (11). The *R*_**U**_(all) column shows the results of comparison when the whole set of motions (librations and vibrations) were used (5). The *R*_**U**_(no V) column indicates the case when only three librations were used while vibration components were excluded (6). The next three columns [*R*_**U**_(*d_x_*, *s_x_*), *R*_**U**_(*d_y_*, *s_y_*) and *R*_**U**_(*d_z_*, *s_z_*)] show the results for cases when only one single libration and a corresponding screw were used (7). The last three columns [*R*_**U**_(*d_x_*), *R*_**U**_(*d_y_*) and *R*_**U**_(*d_z_*)] represent the pure librations (8).

TLS	Method	*R*_**U**_(all)	*R*_**U**_(no V)	*R*_**U**_(*d_x_*, *s_x_*)	*R*_**U**_(*d_y_*, *s_y_*)	*R*_**U**_(*d_z_*, *s_z_*)	*R*_**U**_(*d_x_*)	*R*_**U**_(*d_y_*)	*R*_**U**_(*d_z_*)
PDB entry 2igd									
TLS_CA_	(10)	0.04	0.07	0.14	0.05	0.03	0.00	0.02	0.01
TLS_MC_	(10)	0.09	0.15	0.28	0.01	0.03	0.00	0.02	0.01
TLS_CA_	(11)	0.01	0.02	0.01	0.02	0.04	0.00	0.02	0.01
TLS_MC_	(11)	0.01	0.02	0.03	0.04	0.04	0.00	0.02	0.01
PDB entry 4muy									
TLS_all_	(10)	0.61	0.85	0.89	0.25	0.27	0.01	0.01	0.01
TLS_all_	(11)	0.05	0.11	0.02	0.27	0.42	0.01	0.02	0.00

**Table 2:** Components of the elemental motions The four upper blocks correspond to the TLS matrices for PDB entry 2igd calculated for C^α^ atoms only (TLS_CA_) and for the main-chain atoms (TLS_MC_). The TLS matrices were decomposed with (10) or (11) using the constraints described in Urzhumtsev *et al.* (2015 ▸). The two bottom blocks correspond to the model for PDB entry 4muy. The vectors **v**_*x*_, **v**_*y*_, **v**_*z*_ and **l**_*x*_, **l**_*y*_, **l**_*z*_ of the vibration and libration bases, respectively, are given in Cartesian coordinates in the principal basis [**M**] with the origin at the group centre of mass and with the axes parallel to the crystal axes. The points **w**_*x*_, **w**_*y*_, **w**_*z*_ (in Å) are given in the orthonormal basis [**L**] composed of the principal libration axes **l**_*x*_, **l**_*y*_, **l**_*z*_ and describe the shift of these axes from the origin. The libration amplitudes *d_x_*, *d_y_*, *d_z_* are given in radians and the vibration amplitudes *t_x_*, *t_y_*, *t_z_* and the screw components s*_x_*, *s_y_*, *s_z_* are in Å. For details of the definitions, see Urzhumtsev *et al.* (2013 ▸).

TLS	*t_x_*, *t_y_*, *t_z_*	**v**_*x*_, **v**_*y*_, **v**_*z*_	*d_x_*, *d_y_*, *d_z_*	**l**_*x*_, **l**_*y*_, **l**_*z*_	**w**_*x*_, **w**_*y*_, **w**_*z*_	*s_x_*, *s_y_*, *s_z_*
PDB entry 2igd						
TLS_CA_ (10)	0.163	(−0.085, 0.437, 0.896)	0.011	(−0.262, 0.915, −0.308)	(−12.67, −0.39, 16.71)	−2.07
	0.278	(0.905, 0.410, −0.114)	0.019	(−0.067, 0.301, 0.951)	(1.65, 0.97, 8.55)	−0.88
	0.304	(−0.417, 0.801, −0.430)	0.027	(0.963, 0.270, −0.017)	(−4.67, −3.47, 0.76)	0.80
TLS_MC_ (10)	0.089	(−0.082, 0.334, 0.939)	0.010	(−0.272, 0.943, −0.193)	(−14.16, −1.74, 22.42)	−5.70
	0.277	(0.948, 0.316, −0.030)	0.020	(−0.113, 0.168, 0.979)	(0.49, 0.11, 11.77)	−0.24
	0.314	(−0.306, 0.888, −0.343)	0.027	(0.956, 0.288, 0.061)	(−4.92, −3.54, −0.25)	0.89
TLS_CA_ (11)	0.163	(−0.085, 0.433, 0.897)	0.011	(−0.262, 0.915, −0.308)	(−12.67, −0.39, 16.71)	−0.09
	0.279	(0.902, 0.417, −0.116)	0.019	(−0.067, 0.301, 0.951)	(1.65, 0.97, 8.55)	−0.30
	0.305	(−0.424, 0.799, −0.426)	0.027	(0.963, 0.270, −0.017)	(−4.67, −3.47, 0.76)	1.12
TLS_MC_ (11)	0.083	(−0.078, 0.332, 0.940)	0.010	(−0.272, 0.943, −0.193)	(−14.16, −1.74, 22.42)	−0.43
	0.282	(0.931, 0.362, −0.051)	0.020	(−0.113, 0.168, 0.979)	(0.49, 0.11, 11.77)	0.97
	0.314	(−0.357, 0.871, −0.337)	0.027	(0.956, 0.288, 0.061)	(−4.92, −3.54, −0.25)	1.58
PDB entry 4muy						
TLS_all_ (10)	0.0	(0.951, 0.286, −0.117)	0.001	(0.649, 0.500, −0.573)	(−219.91, −11.67, −256.03)	303.63
	0.257	(−0.220, 0.893, 0.393)	0.008	(−0.633, 0.773, −0.042)	(−49.29, 57.65, −1.63)	2.90
	0.363	(0.216, −0.348, 0.912)	0.014	(0.421, 0.390, 0.819)	(−72.36, −52.48, −124.89)	−3.11
TLS_all_ (11)	0.241	(0.227, 0.947, 0,225)	0.001	(0.649, 0.500, −0.573)	(−219.91, −11.67, −256.03)	0.11
	0.321	(−0.582, −0.053, 0.811)	0.008	(−0.633, 0.773, −0.042)	(−49.29, 57.65, −1.63)	−3.38
	0.396	(0.780, −0.316, 0.540)	0.014	(0.421, 0.390, 0.819)	(−72.36, −52.48, −124.89)	−5.14

**Table 3:** Number of TLS groups with ADP matrices that are reproducible by explicit group motions (*R*_**U**_ ≤ 0.05) PDB content (November 2016): 32 162 entries containing TLS records, 260 353 TLS groups in total. For 263 TLS groups all three matrices were zero and these groups were excluded from further work. Decomposition of TLS matrices into parameters of elemental motions was performed using (9), (10) and (11). The ‘Extracted groups’ column shows the total number of TLS groups for which parameter extraction was possible and ‘Extracted entries’ shows the number of PDB entries for which this was possible for all of the groups. ‘Wrong content’ shows the number of groups for which random-model generation was impossible for technical reasons and ‘Libration undefined’ shows the number of groups for which all libration matrices were zero. Other columns: overall motion (5), overall libration (6), conditions verified for each of the three librations of the group including their screw components (7) and conditions verified for each of the three pure librations of the group (8).

Method	Extracted entries	Extracted groups	Wrong content	Overall motion	Libration undefined	Overall libration	Individual screw	Individual libration
Equation (9)								
Total	4290	88434	314	88120	167	87953	87953	87953
*R*_**U**_ ≤ 0.05				45093		23042	6107	87908
Equation (10)								
Total	4826	95152	332	94820	167	94653	94653	94653
*R*_**U**_ ≤ 0.05				46627		24163	7478	94596
Equation (11)								
Total	4826	95150	332	94818	167	94651	94651	94651
*R*_**U**_ ≤ 0.05				57463		31395	11238	94590

## Footnotes

1 Traditionally, the term *translation* is used to denote the pure shift of the group without rotation. We prefer to use the term *vibration*, which is more coherent when using the term *libration*, and not *rotation*. This allows different components of the **T** matrix to be distinguished: those owing to the proper vibrations and those owing to apparent shifts.

2
**U**_TLS,vibr_ and **U**_ensemble,vibr_ from vibration cancel each other in the numerator of (4) since pure vibrations in the TLS model are always harmonic. Therefore, the denominator of (4) is larger for the full **U** matrices than for the matrices for librations only (this is easier to see in the coordinate system where **U**_TLS,vibr_ is diagonal; the diagonal elements of all **U** matrices are always non-negative). This means that *R*_**U**_ for the overall motion can increase after excluding the vibration component.
